# Rad50 promotes ovarian cancer progression through NF‐κB activation

**DOI:** 10.1111/jcmm.17017

**Published:** 2021-11-03

**Authors:** Yinuo Li, Shourong Wang, Peng Li, Yingwei Li, Yao Liu, Haiya Fang, Xiyu Zhang, Zhaojian Liu, Beihua Kong

**Affiliations:** ^1^ Key Laboratory of Experimental Teratology, Ministry of Education, Department of Obstetrics and Gynecology, Qilu Hospital, Department of Cell Biology, School of Basic Medical Sciences Cheeloo College of Medicine Shandong University Jinan China; ^2^ Advanced Medical Research Institute Cheeloo College of Medicine Shandong University Jinan China; ^3^ Department of Obstetrics & Gynecology Jinhua Hospital of Zhejiang University Jinhua China

**Keywords:** CARD9, NF‐κB, ovarian cancer, Rad50

## Abstract

Rad50 is a component of MRN (Mre11‐Rad50‐Nbs1), which participates in DNA double‐strand break repair and DNA‐damage checkpoint activation. Here, we sought to investigate the clinical and functional significance of Rad50 in high‐grade serous ovarian cancer (HGSOC). We found that Rad50 was frequently upregulated in HGSOCs and enhanced Rad50 expression inversely correlated with patient survival. In addition, ectopic expression of Rad50 promoted proliferation/invasion and induced EMT of ovarian cancer cells, whereas knockdown of Rad50 led to decreased aggressive behaviors. Mechanistic investigations revealed that Rad50 induced aggressiveness in HGSOC via activation of NF‐κB signaling pathway. Moreover, we identified CARD9 as an interacting protein of Rad50 in ovarian cancer cells and the activation of NF‐κB pathway by Rad50 is CARD9 dependent. Our findings provide evidence that Rad50 exhibits oncogenic property via NF‐κB activation in HGSOC.

## 1 INTRODUCTION

Ovarian cancer is the most lethal gynecologic malignancy in women, with an overall 5‐year survival rate of around 30%.^1^ High‐grade serous ovarian carcinoma (HGSOC) is the most common subtype and accounts for up to 70% of all ovarian cancer cases.^2^ HGSOC is usually diagnosed at an advanced stage and exhibits early peritoneal spread and lymph node metastasis, and resistance to conventional chemotherapy.^3^ The development and molecular pathogenesis of HGSOC are largely unknown.

Approximately 50% of HGSOC cases were defective in homologous recombination (HR) DNA repair pathway. BRCA1/2 alterations, which can be derived from a combination of germline and somatic mutations, were seen in 22% of tumours.^4^ Women with germline BRCA1/2 mutations have a 30–70% chance of developing HGSOC by age 70.^5^ Poly (ADP‐ribose) polymerase (PARP) is an abundant nuclear enzyme involved in repairing single‐stand breaks in DNA.^6^ Impaired homologous recombination (HR) DNA repair in cancer cells would render synthetic lethality with PARP1 inhibition.^7^ Olaparib was the first PARP inhibitor approved in the European Union and the United States for the treatment of advanced BRCA‐mutated ovarian cancer.^8^ However, dysfunction of one DNA repair pathway may be compensated for by the function of another DNA damage response (DDR) pathway, which may be augmented and contribute to resistance to DNA‐damaging chemotherapy and radiotherapy.^9^ PARP inhibition in HR‐deficient cells would result in increased activation of DNA‐PK and increased non‐homologous end joining (NHEJ) activity.^10^ High DNA‐PKcs protein expression was associated with advanced stage and correlated with poor ovarian cancer‐specific survival.^11^ Some DNA repair pathways are upregulated in cancer and contribute to the progression of malignancy.^9^

The Mre11‐Rad50‐Nbs1 (MRN) complex plays important roles in the detection and signaling of DNA double‐strand breaks (DSBs), as well as the repair pathways of HR and NHEJ.^12^ MRN complex acts as a double‐strand break sensor for ATM and recruits ATM to broken DNA ends.^13^ The role of the MRN complex in the response to DSBs as well as its requirement for cellular survival makes it a potential target for cancer therapy.^14^ MRE11 promotes tumorigenesis by facilitating resistance to oncogene‐induced replication stress.^15^ Overexpression of NBS1 contributes to transformation through the activation of phosphatidylinositol 3‐kinase/Akt.^16^

Rad50, an ATPase, binds to Mre11 and Nbs1 to form a repair complex that is transported to the nucleus in response to DNA double‐strand breaks.^17^ mRad50 was essential for cellular viability and disruption of mRAD50 causes ES cell lethality, abnormal embryonic development and sensitivity to ionizing radiation.^18^ Frequent lack of RAD50 was found in endometrial^19^ and low‐grade epithelial ovarian cancer,^20^ whereas Rad50 was highly expressed in melanoma^21^ and gastric cancer.^22^ High level of Rad50 was associated with poor prognosis in gastric and colorectal cancer.^22^, ^23^ However, the expression pattern, functional role and clinical implication of Rad50 in HGSOC remain unclear.

In the present study, we found that overexpression of Rad50 was common in HGSOCs and enhanced Rad50 expression inversely correlated with patient survival. Furthermore, we elucidated that Rad50 overexpression promoted cell proliferation and invasiveness potentially via activation of the NF‐κB signaling pathway. Our findings indicate that Rad50 may serve as a potential biomarker and therapeutic target for HGSOC.

## 2 METHODS

### 2.1 Patients and tissue samples

This retrospective study included 151 cases of HGSOC and 33 cases of fallopian tube (FT) which were collected in Qilu hospital from November 2005 to December 2012. The HGSOC specimens were obtained from primary ovarian cancer patients and the FT tissues were from patients who suffered from other benign pathological changes or uterine disease. All participants in this study gave written informed consent as delineated by the protocol which was approved by the Ethics Committee of Shandong University.

### 2.2 Tissue microarray construction and immunohistochemistry

Tissue microarrays (TMAs) were constructed according to the method described previously.^24^ Immunohistochemistry (IHC) staining was performed on formalin‐fixed paraffin‐embedded HGSOC samples and xenograft tumours. Briefly, tissue slides were de‐paraffinized and rehydrated in a graded series of ethanol. After that, antigenic retrieval was proceeded by microwave heating. Nonspecific antigens were blocked with the 1.5% normal goat serum. Then they were incubated at 4°C in a moist chamber overnight with antibodies against human Rad50 (1: 300, Abcam, Cambridge, MA, USA, Cat# ab89) and Ki67 (1:150, DAKO, Tokyo, Japan, Cat# CA626). The next day, the slides were incubated with the corresponding secondary antibody. The staining was detected with a DAB detection system based on the Biotin‐Streptavidin HRP Detection Systems (Zhongshan Biotechnology Company, China). The final score of each sample was assessed by two independent pathologists based on the intensity and extent of staining across the section. Staining intensity was graded according to the following standard: no staining (score 0), weak staining (score 1), moderate staining (score 2) and strong staining (score 3). The proportion of every score was estimated for every sample as 0 (0%), 1 (< 25%), 2 (25–50%), 3 (50–75%) and 4 (> 75%). The final staining score was calculated as the staining intensity score multiplied by the proportion of stained tumour cells. The cases were then divided into low (Score≤6) and high (Score >6) groups.

### 2.3 Cell lines and cell culture

HO8910 cells were obtained from China Type Culture Collection (CTCC, Shanghai, China); A2780, SKOV3 and HEK293T cells were originally purchased from American Type Culture Collection (ATCC, VA, USA). HO8910 and A2780 cells were routinely cultured in RPMI‐1640 medium (Gibco, NY, USA). SKOV3 cells were cultured in McCoy's 5A medium (Gibco, NY, USA) and HEK293T cells were cultured in DMEM (Gibco, NY, USA). All media were supplemented with 10% FBS (Gibco, NY, USA), 100 U/ml penicillin and 100μg/ml streptomycin. All the cells were cultured at 37°C, 5% CO_2_ in a humidified incubator (Thermo Fisher Scientific, IL, USA).

### 2.4 Constructs, lentivirus infection and transient transfection

Rad50 overexpression and knockdown plasmids were obtained from Origene (Origene, MD, USA). Lentivirus was produced in HEK293T cells. For stable infection, 1×10^5^ cells were plated in 6‐well plates with 2 ml medium without antibiotics. After overnight incubation, the medium was replaced by 1 ml Opti‐MEM Reduced‐Serum Medium (Gibco, NY, USA) containing 50 μl of concentrated lentiviral particles and 8 μg/ml of polybrene per well. The fresh medium containing 2 μg/ml of puromycin (Invitrogen, USA) was added to each well 24 hours later and then selected for two weeks. For RNA interference, CARD9 siRNAs and control siRNA were purchased from GenePharma (Shanghai, China) and used for transfecting cells with Lipofectamine 3000 (Invitrogen) according to the manufacturer's protocol. The efficiency CARD9 inhibition was assessed by western blot. All the sequences are listed in Table S2.

### 2.5 Western blot and immunoprecipitation

Western blot and immunoprecipitation were performed as described.^25^ The primary antibodies included anti‐Rad50 (Abcam, USA, Cat# ab89; Santa Cruz, USA, Cat# sc‐74460), anti‐CARD9 (Protein Tech, USA, Cat# 10669–1‐AP; Santa Cruz, USA, Cat# sc‐374569), anti‐p‐p65 (Ser536, Cat# 3033), anti‐p65 (Cat# 8242), anti‐p21^Cip1^ (Cat# 2947), anti‐p27^Kip1^ (Cat# 2947), anti‐Cyclin D1 (Cat# 2978) (Cell Signaling Technology, USA), anti‐GAPDH (Zsbio, China, Cat# TA‐08) and anti‐β‐actin (Sigma‐Aldrich, USA, Cat# A5441). Anti‐E‐cadherin, anti‐N‐cadherin, anti‐Vimentin, anti‐Twist and anti‐Snail antibodies were all from the Epithelial‐Mesenchymal Transition (EMT) Antibody Sampler Kit (Cell Signaling Technology, USA, Cat# 9782).

### 2.6 Cellular proliferation assay

Cell proliferation was assessed by the 3‐(4,5‐dimethylthiazol‐2‐yl)‐2,5‐diphenyltetrazolium bromide (MTT, Sigma‐Aldrich) assay. Cells were plated in 96‐well plates (2–3×10^3^ per well) for continual 1–5 days. After incubation at designated times, 20 μl of MTT (5 mg/ml in PBS) was added to each well, and cells were incubated for another 4 h at 37°C. The supernatants were carefully removed and 200 μl of dimethyl sulfoxide (DMSO, Sigma‐Aldrich) was added to each well. The absorbance of each sample was measured at 490 nm with a Microplate Reader (Thermo Fisher Scientific, IL, USA).

### 2.7 Clonogenic assay

Cells were seeded into 6‐well plates (300–500 cells/well) and cultured for 2–3 weeks. The colonies were fixed with methanol and stained with crystal violet. Colonies containing more than 50 cells were counted. For soft agar colony formation, 3–8×10^3^ cells were suspended in the upper layer (0.7%) of the two layers agar (0.7 and 1.0%) in 6 cm dish. After 3 weeks, the colonies were stained by MTT and the images of colonies were photographed.

### 2.8 Cell cycle analysis

Cells (2‐3x10^6^) were cultured overnight in medium without FBS. Then the cells were cultured in complete medium and harvested at different times. Cell suspensions were incubated with 1ml PBS containing 50 µg/ml PI with RNase A and permeabilization buffer for 30 min. The DNA content was analysed by a flow cytometer with a FASCalibur (BD Biosciences, USA). The results were analysed with the ModFit software (Becton‐Dickinson, USA).

### 2.9 Invasion and migration assay

Invasion and migration assays were performed in 24‐well transwell chambers (BD Biosciences, USA) system with 8μm pores coated with or without diluted matrigel (BD Biosciences, USA). Briefly, 1.5–2×10^5^ cells were seeded into the upper chambers in certain medium containing no FBS, and lower chambers were filled with culture media containing 10–20% FBS as a chemo‐attractant. After incubating at 37 °C for 12 to 48 h depending on the cell lines, cells that penetrated through the membrane were fixed with methanol and stained with crystal violet.

### 2.10 Immunofluorescence

Cells were seeded on sterile glass coverslips and cultured overnight at 37℃. Cells were washed with PBS and then fixed for 20min with 4% paraformaldehyde. Following blocking with 10% goat serum in PBS for 2 h at room temperature, the cells were incubated with primary antibodies (anti‐N‐cadherin, anti‐E‐cadherin and anti‐p65) overnight at 4℃. Cells were then incubated with rhodamine/FITC‐labelled secondary antibody (Beyotime Biotechnology, China) for 1 h at room temperature in the dark. Cells were washed with PBS three times before counterstaining with 1 µg/ml DAPI for 5 min in the dark. Finally, the slides were mounted with anti‐fading reagent and examined with a fluorescence microscope (Olympus, Japan).

### 2.11 Xenograft tumour model

Female BALB/c nude mice (aged 4‐ 6weeks) were injected subcutaneously in bilateral flanks with cell suspensions (5×10^6^ cells in 100 µl PBS). Tumour size was measured every three days after 1 week, and mice were killed with anaesthesia after three weeks. Tumour volume was calculated according to the formula TV (cm^3^) =a × b^2^ × π/6, where ‘a’ is the longest diameter, and ‘b’ is the shortest diameter. For the 2 in vivo nude mice metastasis assays, 150 µl suspensions of 5×10^6^ cells were used for intraperitoneal injection and cells (2×10^6^ in 100 µl PBS) were injected into the lateral tail veins. After 5 or 7 weeks, the mice were killed under anaesthesia. Mice injected intraperitoneally were examined for the presence and extent of intra‐abdominal tumours. The lungs from the mice injected through tail veil were collected and fixed in 10% formalin. For tissue morphology evaluation, haematoxylin and eosin (H/E) staining was performed on sections from embedded samples. The metastatic nodules were counted on each H&E section under an optical microscope and the microscopic counting was taken as the final number of lung metastatic nodules. All animal experiments were performed with the approval of Shandong University Animal Care and Use Committee.

### 2.12 Statistical analysis

The software GraphPad Prism 5 was used for statistical analysis. The log rank test was used to compare the overall survival for HGSOC patients dichotomized based on low (Score ≤6) versus high (Score >6) Rad50 IHC expression level. The two‐tailed Student's t‐test and one‐way ANOVA analysis were used to analyse the differences. The relationship between the expression of Rad50 and clinical pathological factors was analysed by the chi‐squared test or Fisher's exact test. All error bars represent the standard error of three separate experiments. Differences with *p* < 0.05 were considered significant.

## 3 RESULTS

### 3.1 Rad50 is commonly overexpressed in HGSOCs and correlates with poor prognosis

**FIGURE 1:**
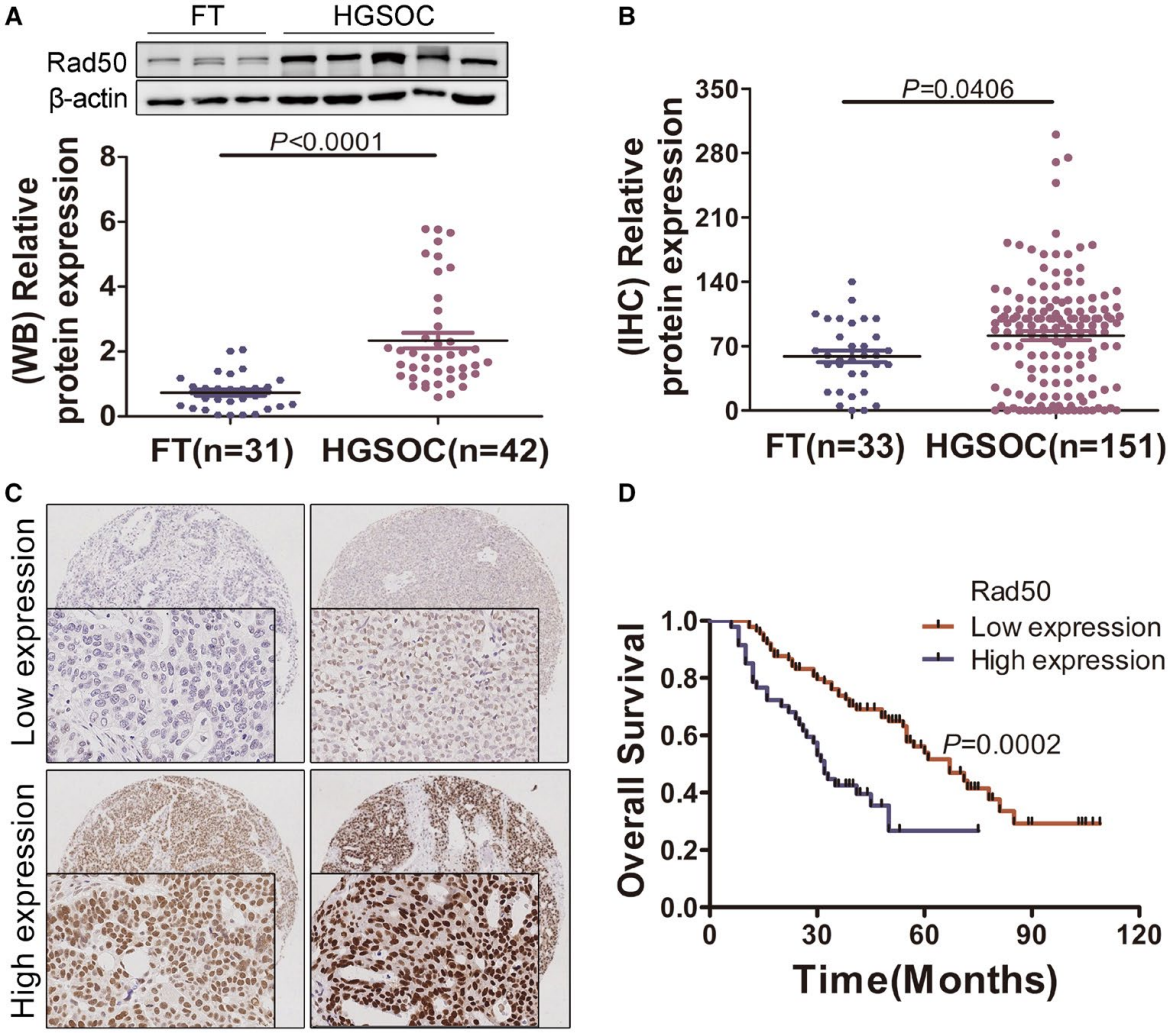
Rad50 is commonly elevated in HGSOCs and correlates with poor prognosis. (A) Western blot analysis of Rad50 in HGSOC (n=42) compared to normal fimbria (n=31). (B) The plot graph reveals the statistical result of Rad50 expression in HGSOC (n=151) compared to normal fimbria (n=33) assessed by IHC. *p < 0.05, **p<0.01, ***p<0.001, (Student's t‐test). (C) Representative images of immunohistochemical staining of Rad50 in the tissue microarray. (D) Kaplan–Meier plots of overall survival for HGSOC patients dichotomized based on low (Score ≤6) versus high (Score >6) Rad50 IHC expression level (Log rank test, *p*=0.0002)

To determine the expression and clinical significance of Rad50 in HGSOCs, we first examined Rad50 expression by western blot in HGSOCs and in fallopian tube (FT). As shown in Figure 1A and Figure S1A, Rad50 was overexpressed in a large proportion of the HGSOCs (n=42) compared to FTs (n= 31). Consistently, immunohistochemical staining revealed a significant increase of Rad50 level in HGSOCs (n=151) compared with FTs (n=33) as illustrated in Figure 1B. Strikingly, Rad50 was increased in olaparib‐resistant ovarian cancer cells in GSE 117765 data (Figure S1B). Clinical Proteomic Tumour Analysis Consortium (CPTAC) data also revealed upregulation of Rad50 in ovarian, breast, colon cancers and Clear cell renal cell carcinoma (Figure S1C).^26^ Additionally, we analysed clinicopathological and prognostic significance of Rad50 expression level in HGSOCs (Table S1). Elevated Rad50 expression was correlated with lymph nodes metastasis (*p*=0.0410) and the omentum metastasis (*p*=0.0365). The association between Rad50 expression and prognosis was analysed by Kaplan–Meier survival analysis. As shown in Figure 1C,D, the high Rad50 level was significantly correlated with short overall survival (*p*=0.0002). These findings suggest that Rad50 is commonly increased and upregulation of Rad50 is associated with progression and poor prognosis in patients with HGSOC.

### 3.2 Rad50 promotes proliferation and anchorage‐independent growth in ovarian cancer cells

**FIGURE 2:**
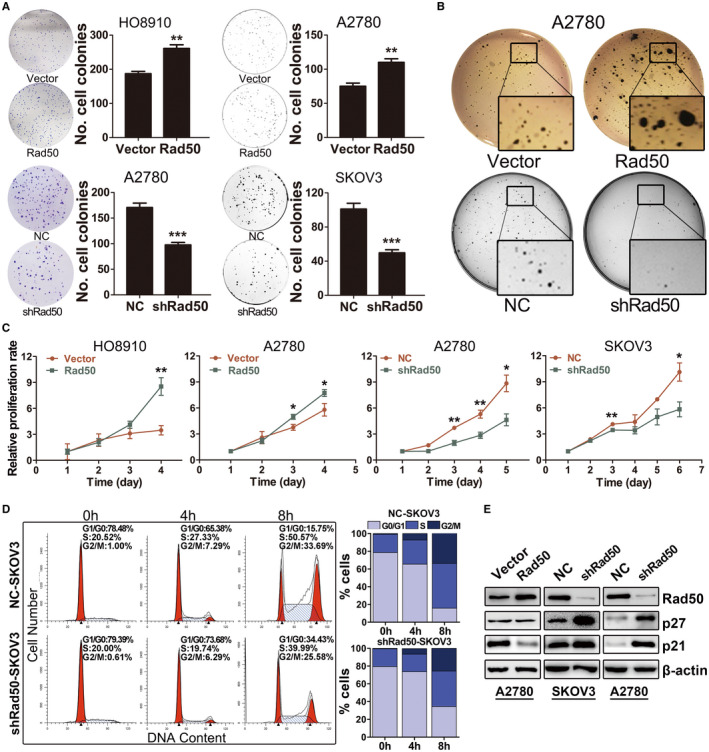
Rad50 promotes proliferation and anchorage‐independent growth in ovarian cancer cells. (A) Effect of Rad50 on clonogenic ability in ovarian cancer cell lines. (B) Soft agar colony formation assay exhibited the effect of Rad50 on anchorage‐independent growth and malignant transformation. (C) Growth curves illustrated the cell proliferation in A2780, SKOV3 and HO8910 cells. (D) Cell cycle analysis of synchronized SKOV3 cells with Rad50 knockdown in comparison with control cells measured by flow cytometry after 0, 4‐ and 8‐hours culture in the complete medium. (E) Western blot analysis of cell cycle–related proteins in ovarian cancer cell lines with Rad50 overexpression or knockdown. The data shown here are from a representative experiment repeated three times with similar results. Data are presented as means ±S.D. *p < 0.05, **p<0.01, ***p<0.001, when compared with the control group (Student's t‐test)

To determine the function of Rad50 in ovarian cancer, we established cell lines with Rad50 overexpression (Rad50‐OVE) or knockdown (shRad50) as illustrated in Figure S1D. We next performed clonogenic assay to evaluate the effect of Rad50 on ovarian cancer cells. High expression of Rad50 enhanced clonogenic capacity in HO8910 and A2780 cells, whereas depletion of Rad50 significantly reduced the colony‐forming efficiency in A2780 and SKOV3 cells (Figure 2A). We further conducted soft agar colony formation assay and found that Rad50 overexpression significantly increased the number and size of colonies in soft agar (Figure 2B), exhibiting more malignant transformation ability. Moreover, growth curve analysis showed that forced Rad50 expression promoted cell proliferation, whereas Rad50 knockdown significantly suppressed cell proliferation compared with control cells (Figure 2C). After overnight serum starvation, we replaced the media with complete media and harvested the cells at the indicated time points. Cell cycle analysis revealed that Rad50 knockdown decreased the percentage of cells at S and G2‐M phases relative to control group (Figure 2D and Figure S2A). Accordingly, the protein levels of p21 and p27 were notably increased in A2780 and SKOV3 cells with Rad50 knockdown (Figure 2E). These results indicate that Rad50 promotes proliferation and clonogenic capacity of ovarian cancer cells.

### 3.3 Rad50 promotes ovarian cancer cell invasion and induces epithelial‐mesenchymal transition (EMT)

**FIGURE 3:**
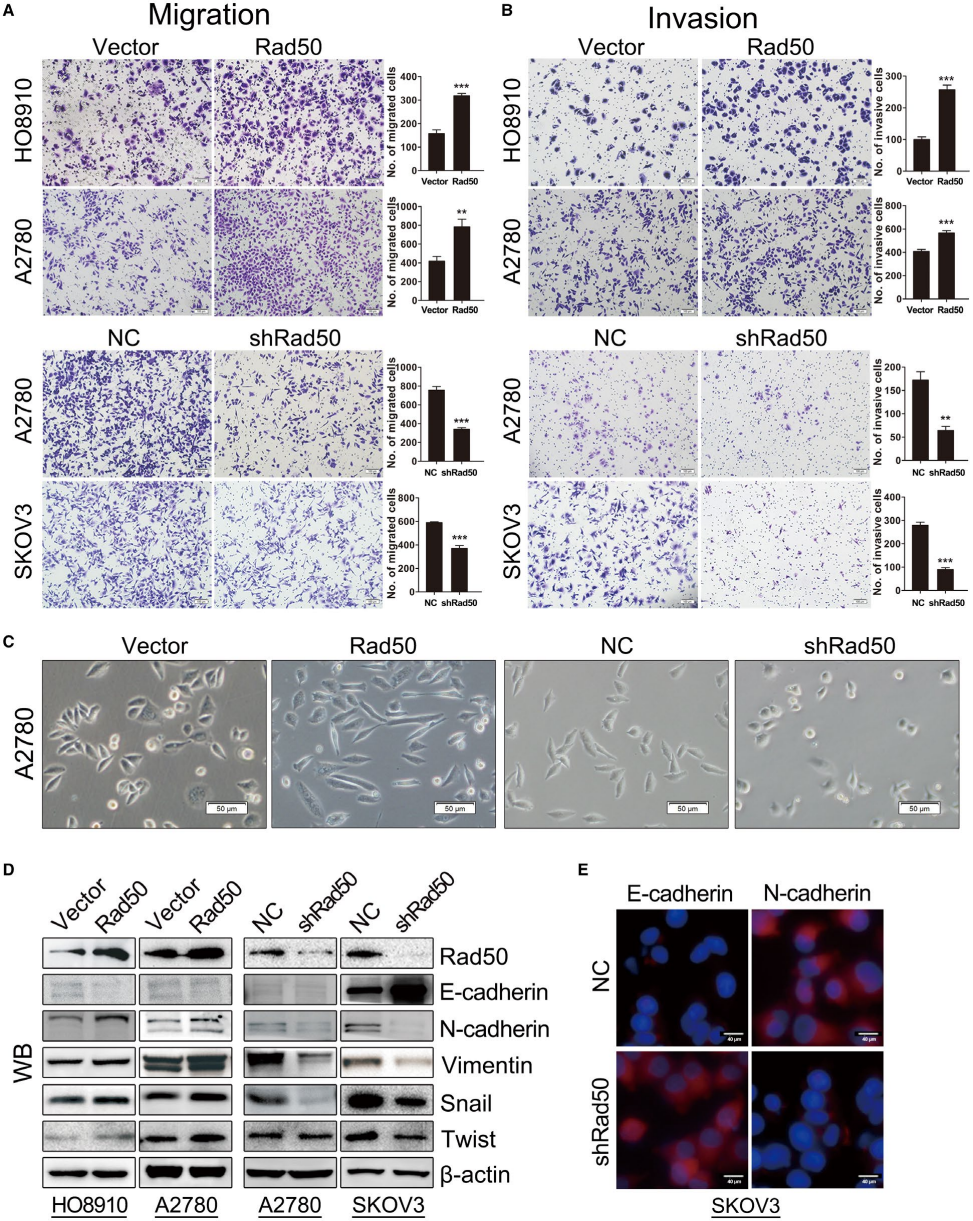
Rad50 promotes migration, invasion and induces EMT of ovarian cancer cells in vitro. (A and B) Migration and invasion were performed in A2780, SKOV3 and HO8910 cells with Rad50 overexpression or knockdown. (C) Morphological changes of A2780 cells with Rad50 overexpression or knockdown. (D) EMT‐related markers were detected by western blot in A2780, SKOV3 and HO8910 cells with Rad50 overexpression or knockdown. (E) Immunofluorescence staining showed E‐cadherin and N‐cadherin expression in SKOV3 cells with Rad50 knockdown. **p<0.01, ***p<0.001, when compared with the control group (Student's t‐test)

Because higher level of Rad50 in HGSOC patients was significantly related to metastasis, we hypothesized that Rad50 promotes invasion capacity of ovarian cancer cells. We then tested migration and invasion abilities by transwell assay. A2780 and HO8910 cells with Rad50 overexpression significantly increased the migration and invasion potential while knockdown of Rad50 in A2780 and HO8910 cells exhibited reduced migration and invasion ability relative to control cells (Figure 3A,B). Meanwhile, we found that HO8910 and A2780 cells with Rad50 overexpression exhibited more spindle‐like morphology while knockdown Rad50 in A2780 and SKOV3 cells exhibited a more epithelial morphology compared to control cells (Figure 3C). We further assessed the expression of EMT markers by western blot and immunofluorescence. As shown in Figure 3D,E, forced expression of Rad50 in A2780 and HO8910 cells increased the expression of mesenchymal phenotype markers (N‐cadherin, Vimentin, Snail, Twist) and reduced the expression of epithelial phenotype marker (E‐cadherin) while Rad50 depletion had the opposite effect. Quantified data are shown in Figure S3. These data suggest that Rad50 promotes migration /invasion and induces EMT in ovarian cancer cells.

### 3.4 Rad50 promotes tumour growth and metastasis of tumour xenografts

**FIGURE 4:**
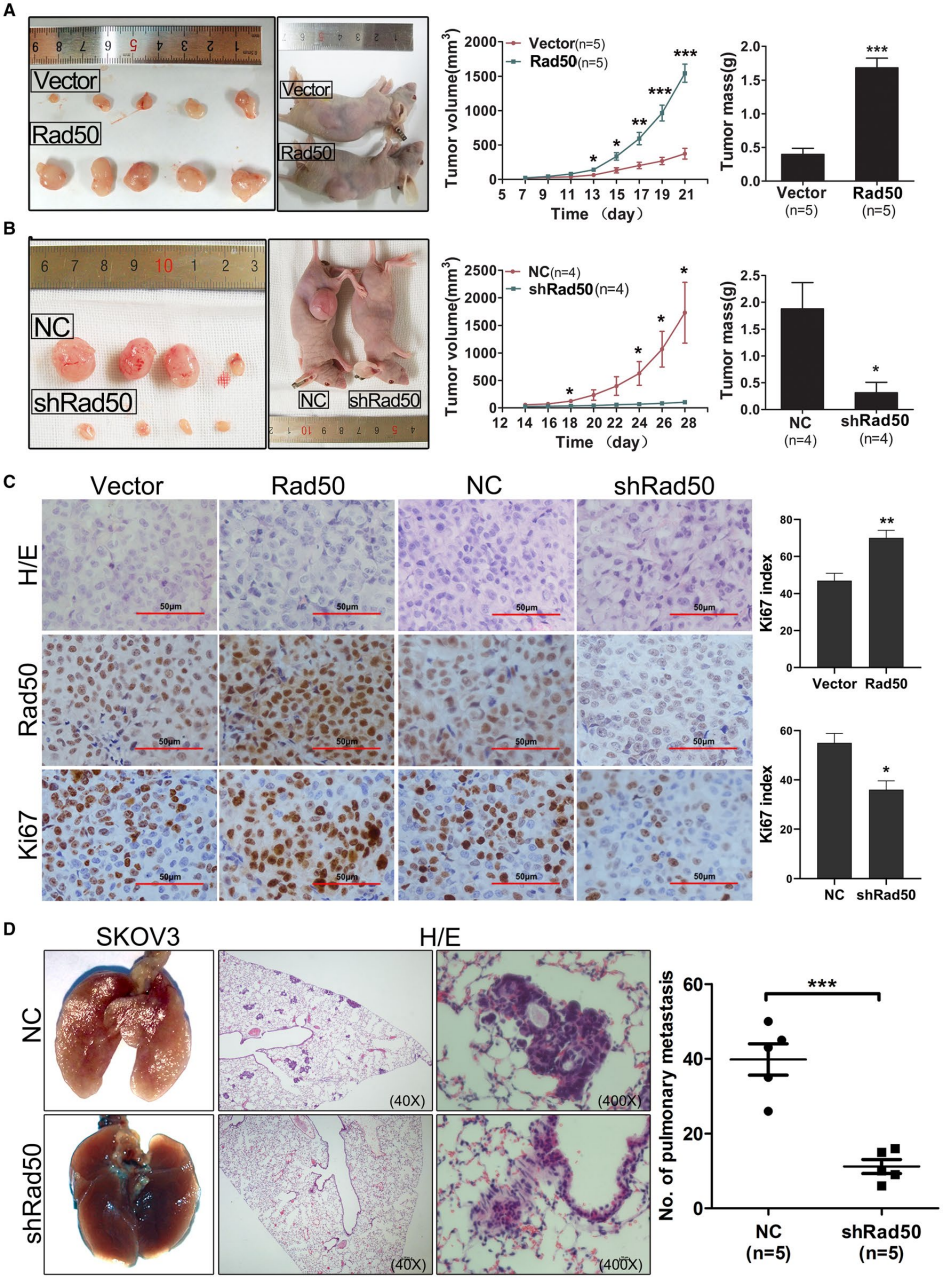
Rad50 promotes tumour growth and metastasis of xenografts. (A‐B) Photographs illustrated tumours in xenografts via subcutaneous injection of A2780 cells with Rad50 overexpression or knockdown. (C) Representative images of HE staining and IHC staining with indicated antibodies of tumours isolated from mice. (D) Representative images of lungs and HE staining isolated from mice that received tail vein injection. *p < 0.05, **p<0.01, ***p<0.001, student's t‐test was performed when comparing 2 groups, one‐way ANOVA was performed when comparing multiple groups

We next assessed the potential role of Rad50 on tumour growth and metastasis *in vivo*. Overexpression of Rad50 in A2780 cells significantly promoted ovarian tumour growth (Figure 4A), whereas knockdown of Rad50 in SKOV3 cells remarkably reduced tumour burden in mice (Figure 4B). Immunostaining of Ki‐67 showed a higher proliferative index in tumours with Rad50 overexpression and a lower proliferative index in tumours with Rad50 knockdown than their corresponding control cells (Figure 4C). We further evaluated the metastasis‐promoting potential of Rad50 in the lung metastasis model via tail vein injection and in the intraperitoneal metastatic model via intraperitoneal injection. As shown in Figure 4D, knockdown of Rad50 in SKOV3 cells significantly reduced the number and size of lung metastasis nodules compared with control. Meanwhile, overexpression of Rad50 in A2780 cells aggregated and formed much more disseminated peritoneal tumours, whereas no peritoneal tumour was observed in the control group (Figure S4). These results strongly suggest that Rad50 possesses potent oncogenic properties *in vivo*.

### 3.5 Rad50 induces aggressiveness in HGSOC via activation of NF‐κB signaling pathway

**FIGURE 5:**
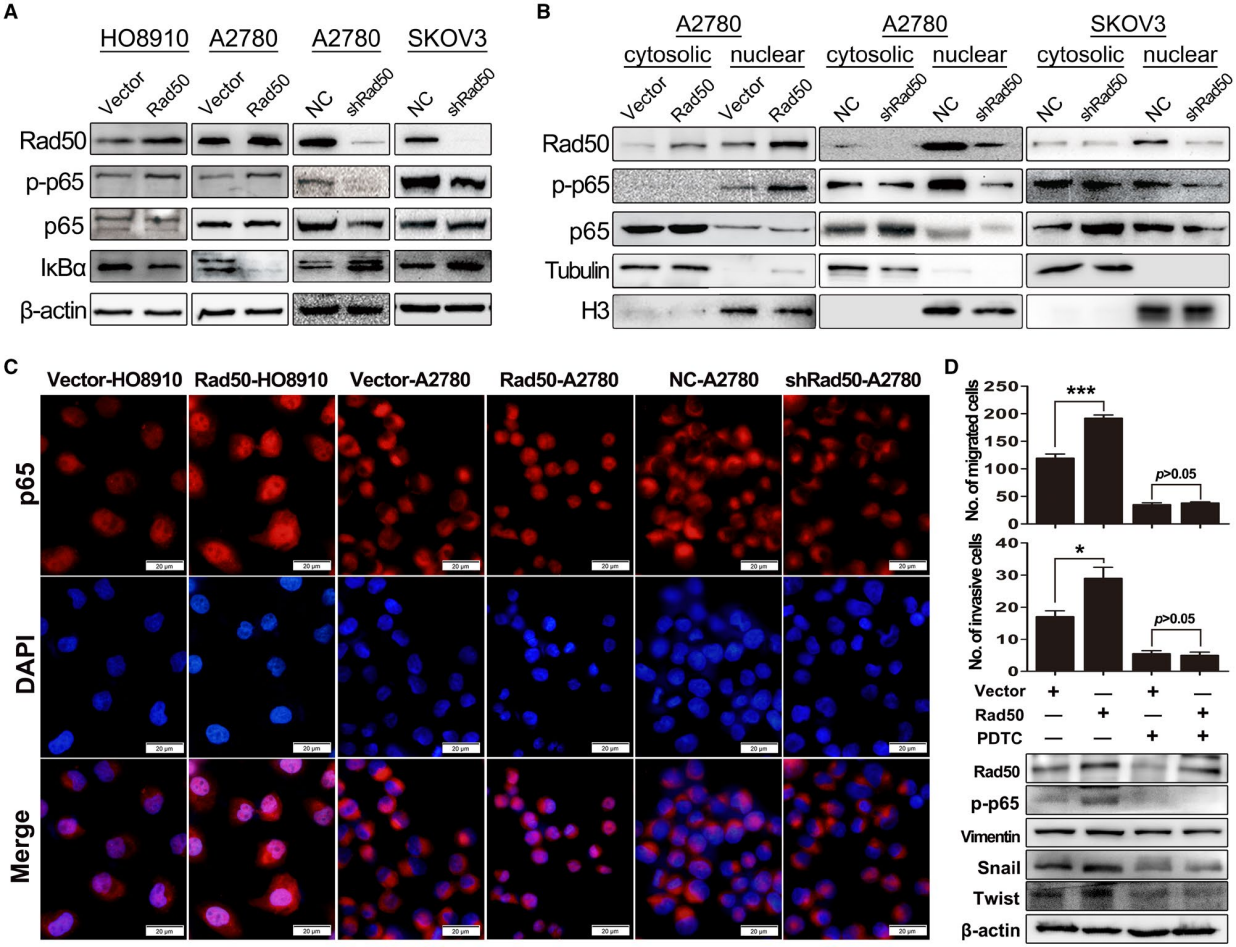
Rad50 induces aggressiveness in HGSOC via activation of NF‐κB signaling pathway. (A) Western blot analysis showing the expression of p65, p‐p65 and IκBα in ovarian cancer cell lines with Rad50 overexpression or knockdown. (B) Western blot analysis of indicated proteins in cytoplasmic and nuclear extracts from A2780 and SKOV3 cells with Rad50 overexpression or knockdown. (C) Immunocytofluorescence showing cytoplasmic or nuclear localization of the NF‐κB complex in the indicated cell lines. (D) In the absence or presence of NF‐κB inhibitor PDTC, migration/invasion ability and NF‐κB downstream targets were measured with or without Rad50 overexpression. *p < 0.05, **p<0.01, ***p<0.001, one‐way ANONA

A study on dendritic cells revealed that Rad50‐CARD9 interaction activates NF‐κB pathway when transfected with dsDNA or infected a DNA virus.^27^ We then postulated that Rad50 may activate NF‐κB pathway in ovarian cancer. We first examined the NF‐κB pathway via immunoblot. Interestingly, overexpression of Rad50 increased the phosphorylation of p65 and decreased total IκBα protein levels while knockdown of Rad50 significantly decreased p‐p65 protein level in ovarian cancer cell lines (Figure 5A). We further examined the nuclear localization of p65 protein by western blot of fractionated proteins and found that levels of the activated nuclear form p‐p65 were significantly higher in A2780 cells with Rad50 overexpression compared to control cells (Figure 5B, left panel), whereas knockdown of Rad50 inhibited the translocation of p‐p65 to the nucleus in both A2780 and SKOV3 cell lines (Figure 5B, middle and right panels). Immunofluorescence data showed that ectopic expression of Rad50 induced, whereas downregulation of Rad50 inhibited nuclear localization of p‐p65 (Figure 5C). To determine whether Rad50 drives the HGSOC aggressive behaviour through NF‐κB activation, we blocked the NF‐κB pathway by PDTC in Rad50‐overexpressing cells. As expected, PDTC treatment decreased Rad50‐induced invasiveness and EMT (Figure 5D and Figure S5A). These results suggest that Rad50 activates NF‐κB and the oncogenic function of Rad50 is dependent on the activation of NF‐κB.

**FIGURE 6:**
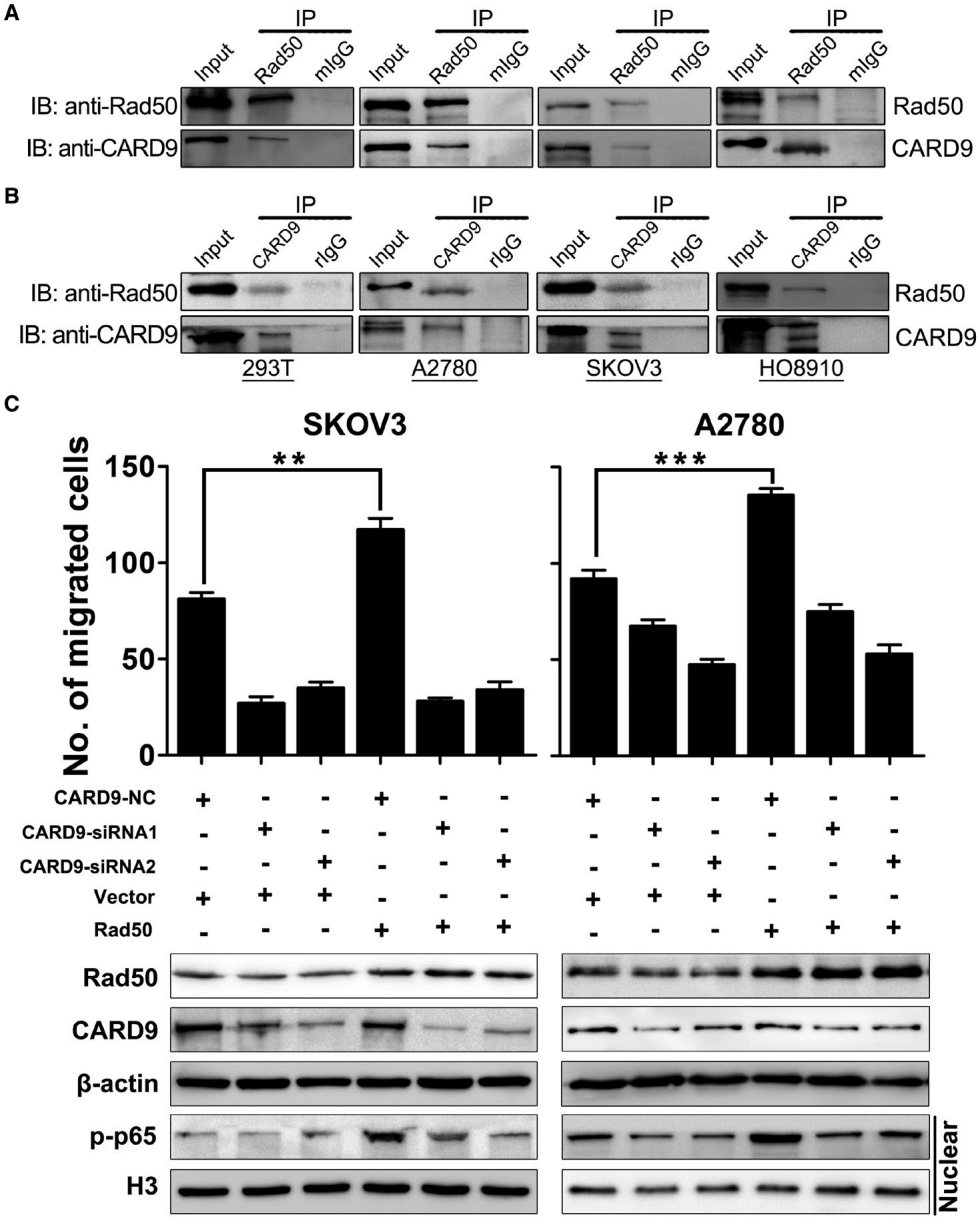
Rad50‐mediated activation of NF‐κB pathway is CARD9 dependent. (A) Interaction of Rad50 and CARD9 was determined by co‐IP and western blot in HEK293T and ovarian cancer cell lines immunoprecipitated with either anti‐ Rad50 antibody or IgG control. (B) Interaction of Rad50 and CARD9 was determined by co‐IP and western blotting in indicated cell lines immunoprecipitated with anti‐CARD9 antibody or IgG control. (C) Transwell and western blot analysis of SKOV3 and A2780 cells transfected with indicated combinations of siRNA or vectors. *p < 0.05, **p<0.01, ***p<0.001, one‐way ANONA

We proposed that Rad50 may also activate NF‐κB pathway via the interaction with CARD9 in ovarian cancer cells. To this end, we performed immunoprecipitation (IP)–western blot to test whether Rad50 can interact with CARD9 in ovarian cancer cells. As indicated in Figure 6A, CARD9 was detected in the immunoprecipitates with anti‐Rad50 antibody, but not with the control mouse IgG in HEK293T and ovarian cancer cell lines. Consistently, Rad50 was detected in the immunoprecipitates with anti‐CARD9 antibody (Figure 6B). To verify whether Rad50‐mediated activation of NF‐κB pathway is CARD9 dependent, we knocked down CARD9 in Rad50‐overexpressed ovarian cancer cell lines and found that knockdown of CARD9 significantly reversed Rad50‐induced migration activities (Figure S5B). Consistent with this, activated nuclear form p‐p65 induced by Rad50 was attenuated by siRNAs targeting CARD9 (Figure 6C). Therefore, we identified CARD9 as an interacting protein of Rad50 in ovarian cancer cells and Rad50‐mediated activation of NF‐κB pathway is CARD9 dependent.

## 4 DISCUSSION

DDR is a double‐edged sword in cancer prevention and cancer therapy. It acts as a positive guardian of genomic stability to prevent tumorigenesis. On the other hand, DDR also functions as a negative saboteur to resist chemo‐ and radiotherapy.^28^ In our study, we reported that MYC proto‐oncogene transcriptionally activated Rad50 expression in HGSOC. Rad50 was commonly upregulated in HGSOC and enhanced Rad50 expression inversely correlated with patient survival and metastasis. In agreement with our results, previous studies also reported that high expression of Rad50 was associated with poor prognosis in lung^29^ and gastric^22^ cancer.

In addition to conferring chemoresistance and radioresistance, high expression of DNA repair genes has been shown to exhibit oncogenic properties. Overexpression of Ku proteins promotes oncogenic phenotypes, including hyperproliferation and resistance to apoptosis.^30^ DNA‐PKcs drive prostate cancer progression and metastasis via transcriptional regulation.^31^ MRE11 promotes cell proliferation, tumour invasion and DNA repair in breast cancer.^32^ Increased NBS1 expression is a marker of aggressive head and neck cancer and overexpression of NBS1 contributes to transformation.^33^ PARP1, which is overexpressed in ovarian cancer, was shown to promote cancer cell survival by repressing the expression of NOX 1 and NOX4.^34^ Our findings in this study demonstrated that Rad50 has oncogenic properties in ovarian cancer. These studies expanded our understanding of the functions of DNA repair genes in tumorigenesis.

Although Rad50‐CARD9 interaction activates NF‐κB pathway that has been reported in dendritic cells,^27^ we demonstrated that Rad50‐CARD9 interaction activates NF‐κB pathway in ovarian cancer cells. Overexpression of Rad50 increased the phosphorylation of p65 and decreased total IκBα protein levels while knockdown of Rad50 significantly decreased p‐p65 protein level in ovarian cancer cell lines. PDTC treatment decreased Rad50‐induced invasiveness and EMT. Furthermore, we verified CARD9 as a specific interacting protein of Rad50 in ovarian cancer cells and Rad50‐mediated activation of NF‐κB pathway is CARD9 dependent. Many human cancers, including ovarian cancer, possess high levels of constitutive NF‐κB activity.^35^ CARD9 contributes to tumour metastasis by promoting metastasis‐associated macrophage polarization through activation of the NF‐κB signaling pathway.^36^ Thus, it is possible that ectopic expression of Rad50 may recruit CARD9 and contribute to tumour metastasis through activating NF‐κB pathway in HGSOC. Future studies will be required to explore the molecular cooperation between Rad50 and CARD9 in promoting proliferation/invasion and inducing EMT in ovarian cancer.

In summary, our findings showed that Rad50 exhibits oncogenic property in ovarian cancer. We provided evidence that Rad50 ectopic expression promotes migration/invasion and induces EMT in ovarian cancer cells. Rad50 induces aggressiveness in HGSOC via activation NF‐κB signaling pathway. Our data suggest that Rad50 could be a prognostic biomarker and a potential therapeutic target for ovarian cancer.

## CONFLICT OF INTEREST

No potential conflicts of interest were disclosed.

## AUTHOR CONTRIBUTIONS

**Yinuo Li:** Conceptualization (equal); Data curation (equal); Formal analysis (equal); Methodology (equal); Writing‐original draft (equal). **Shourong Wang:** Data curation (equal); Formal analysis (equal); Methodology (equal). **Peng Li:** Conceptualization (equal); Data curation (equal); Resources (equal). **Yingwei Li:** Data curation (equal); Formal analysis (equal); Investigation (equal). **Yao Liu:** Data curation (equal); Formal analysis (equal). **Haiya Fang:** Conceptualization (equal); Data curation (equal); Methodology (equal). **Xiyu Zhang:** Conceptualization (equal); Data curation (equal); Methodology (equal). **Zhaojian Liu:** Conceptualization (equal); Funding acquisition (equal); Investigation (equal); Methodology (equal); Project administration (equal); Supervision (equal); Writing‐review & editing (equal). **Beihua Kong:** Conceptualization (equal); Investigation (equal); Project administration (equal).

## Supporting information

Fig S1‐S5

Table S1‐S2

Supplementary Material

Supplementary Material
