# Supplementary material for: Rad50 promotes ovarian cancer progression through NF‐κB activation
Source: J Cell Mol Med. 2021 Nov 3;25(23):10961–72. doi: 10.1111/jcmm.17017 (PMC8642684; doi:10.1111/jcmm.17017)

Supplementary Figure 1

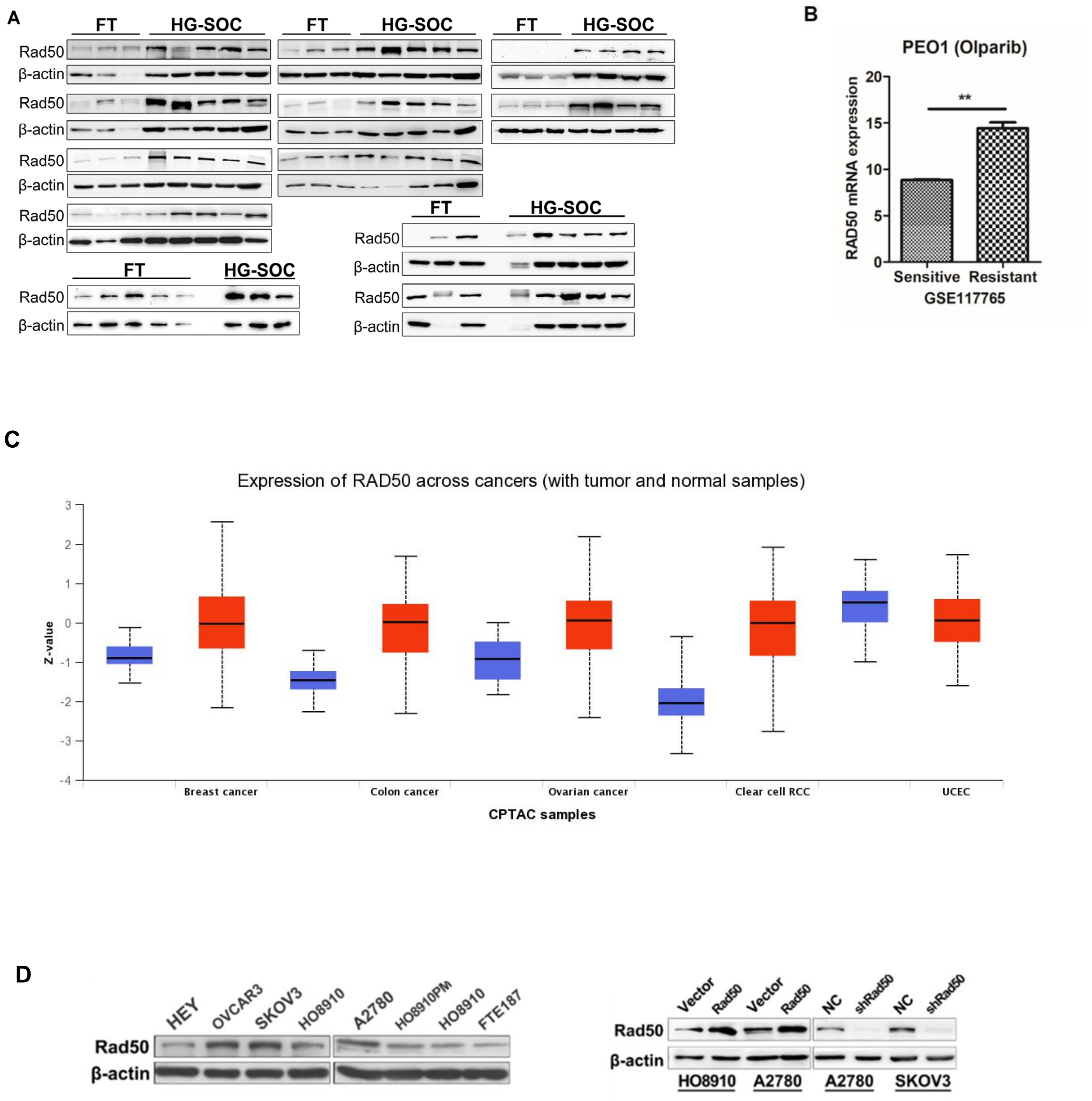

Supplementary Figure 2

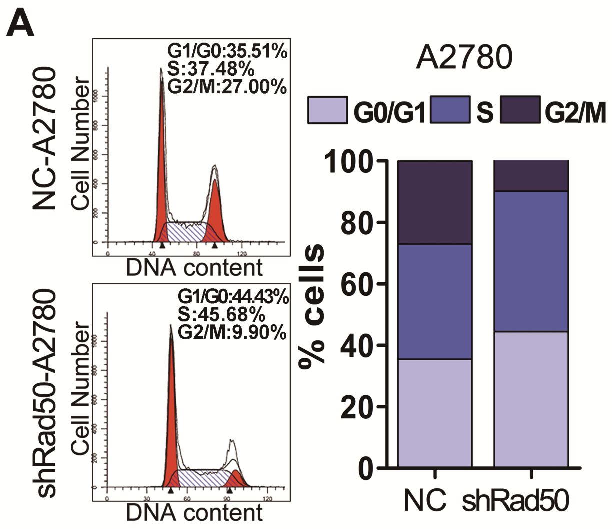

Supplementary Figure 3

A

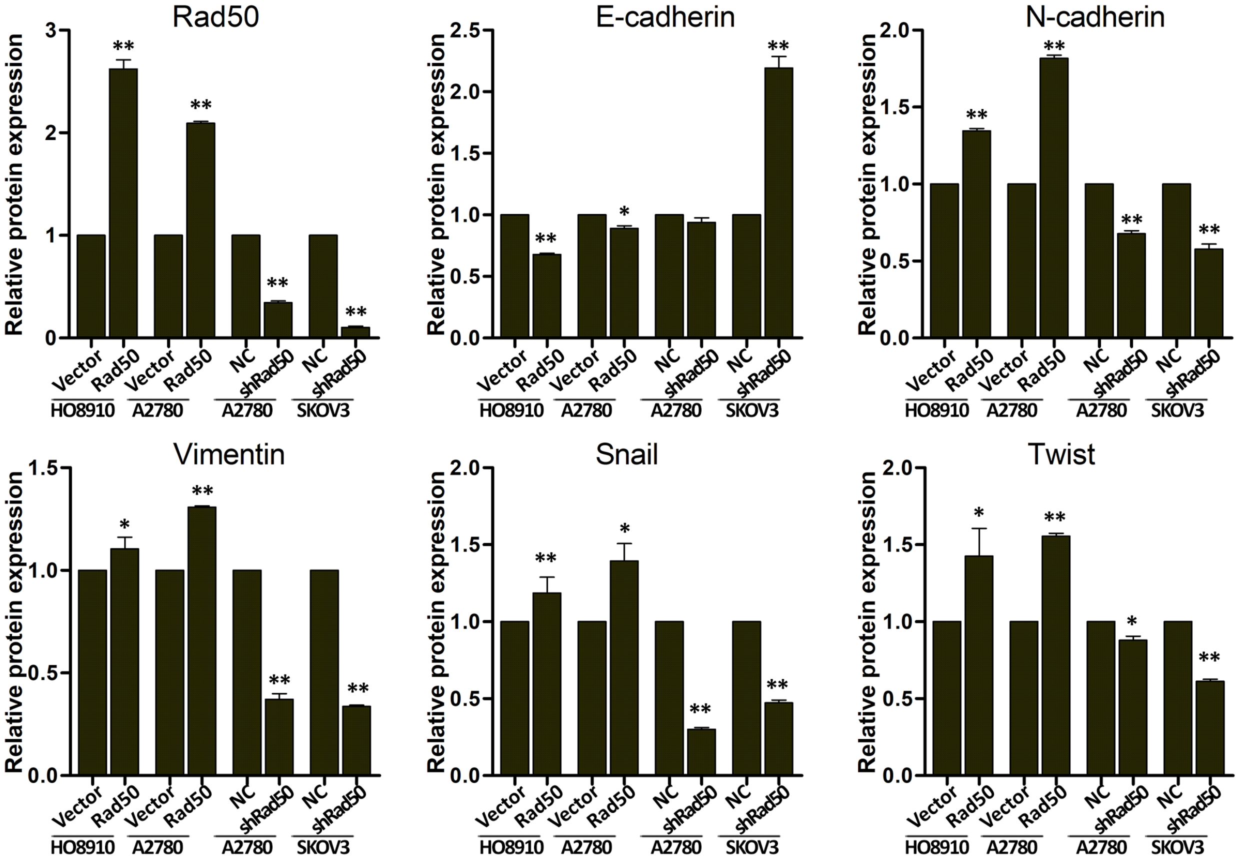

Supplementary Figure 4

A

Vector-A2780

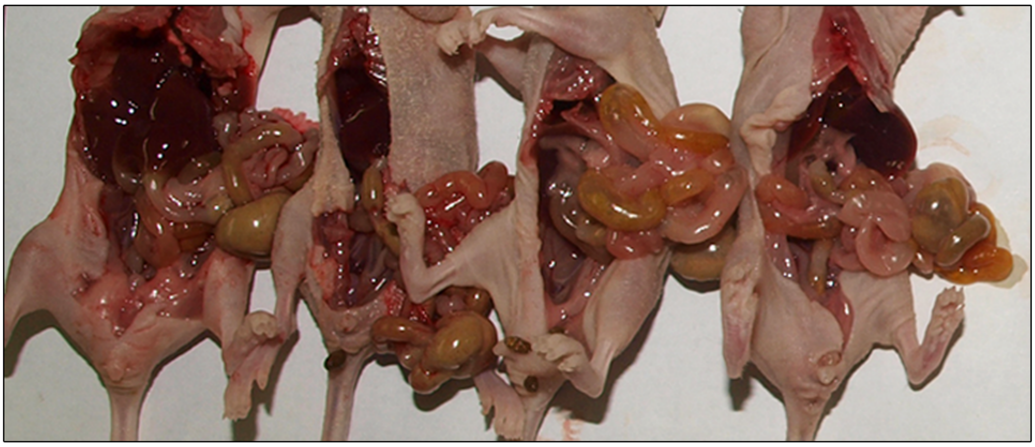

Rad50-A2780

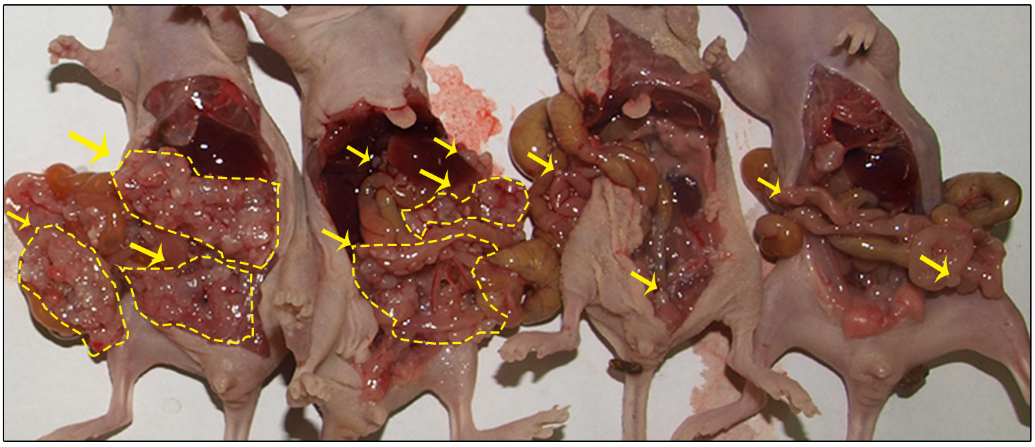

Supplementary Figure 5

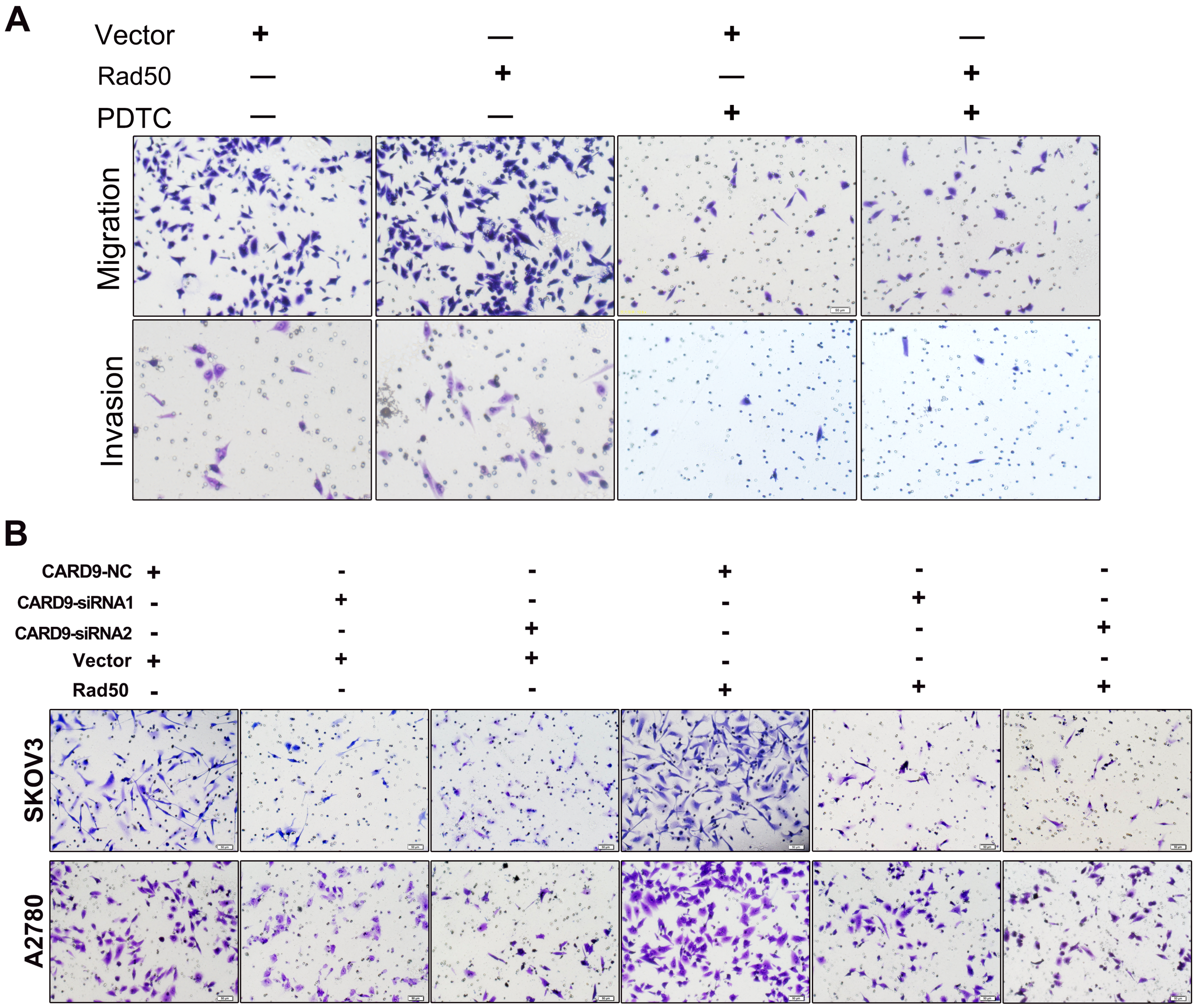

Supplement: Supplementary file 1 — Fig S1‐S5 [file JCMM-25-10961-s004.pdf]
