# Supplementary material for: Rad50 promotes ovarian cancer progression through NF‐κB activation
Source: J Cell Mol Med. 2021 Nov 3;25(23):10961–72. doi: 10.1111/jcmm.17017 (PMC8642684; doi:10.1111/jcmm.17017)
Supplement: Supplementary file 2 — Table S1‐S2 [file JCMM-25-10961-s002.docx]

| **Table S1**  Correlation between Rad50 expression and clinicopathological characteristics | | | |
| --- | --- | --- | --- |
|  | | | |
| **Clinicopathological Variables** | **Number** | **Rad50 expression**  **Low level High level** | ***P* value ^a^** |
| **Age (years)** |  |  |  |
| <56 | 73 | 43 30 | 0.1230 |
| ≥56 | 78 | 56 22 |  |
| **Stage** |  |  |  |
| Early (stagesⅠ-Ⅱ) | 36 | 27 9 | 0.2282 |
| Advanced (stages Ⅲ-Ⅳ) | 115 | 72 43 |  |
| **Lymph nodes metastasis** |  |  |  |
| Negative | 25 | 21 4 | 0.0410 |
| Positive | 28 | 16 12 |  |
| **Omentum metastasis** | | | |
| Negative | 81 | 61 20 | 0.0365 |
| Positive | 70 | 41 29 |  |
| **CA 125 in serum (U/mL)** |  |  |  |
| <600 | 61 | 44 17 | 0.2217 |
| ≥600 | 90 | 55 35 |  |
| ^a^ Independent-samples t-test. | | | |

**Table S2**

Primers and siRNA or shRNA sequences used in this study

| Method | Name | Sequence (5 ′—3 ′) |
| --- | --- | --- |
| si-RNA | si-CARD9-1 | GGGUAGAGCAAGACAAGUU |
| si-RNA | si-CARD9-2 | CCAACUAUGAAGAGUCCAU |
| sh-RNA | sh-Rad50 | CGCCUAAAGAACGACAUAGAA |
| si-RNA | Negative control | UUCUCCGAACGUGUCACGU |
