# Supplementary material for: Rad50 promotes ovarian cancer progression through NF‐κB activation
Source: J Cell Mol Med. 2021 Nov 3;25(23):10961–72. doi: 10.1111/jcmm.17017 (PMC8642684; doi:10.1111/jcmm.17017)

Raw data of Western blots index for Figure 1A and Supplementary Figure 1A

The raw data files were named according to red index here.

Figure 1A

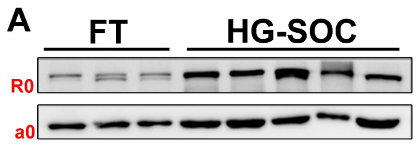

Revised Supplementary Figure 1A

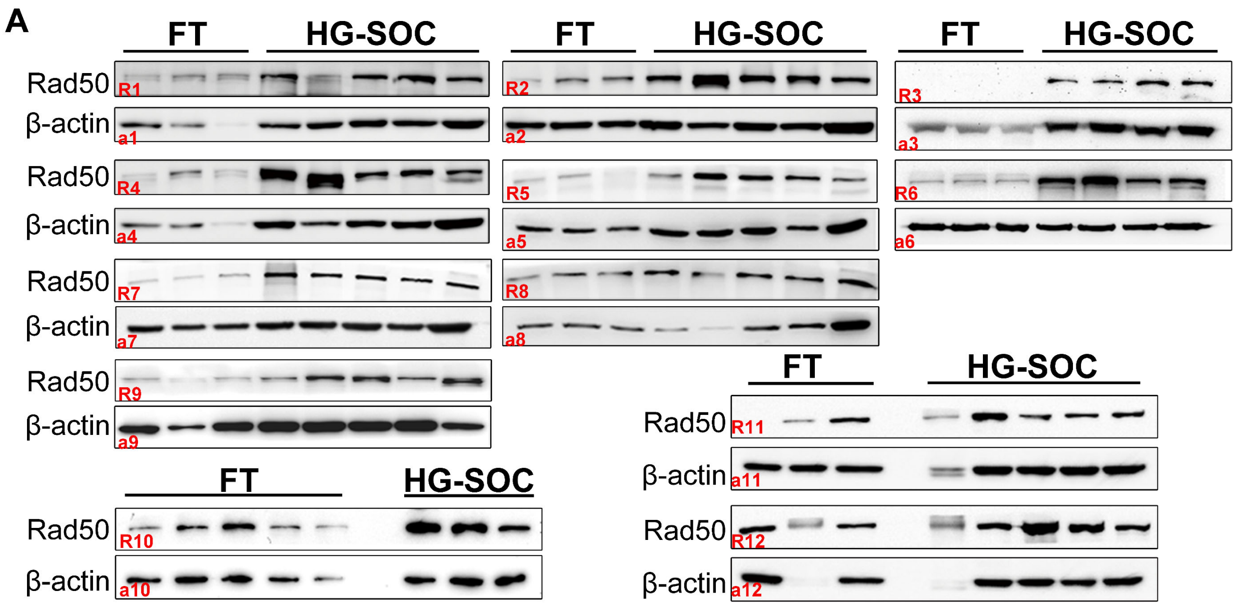

Raw data of Western blots of Rad50

To save antibody and PVDF membrane, we cropped the membrane when we blotted with antibody.

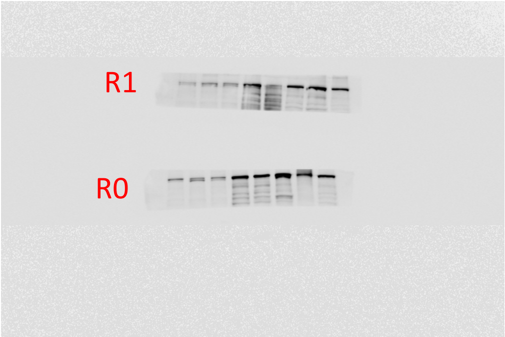

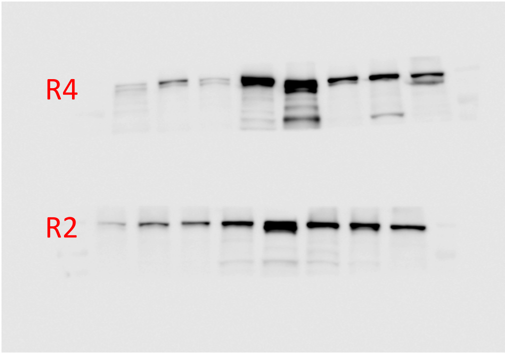

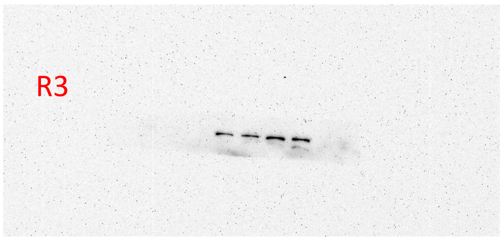

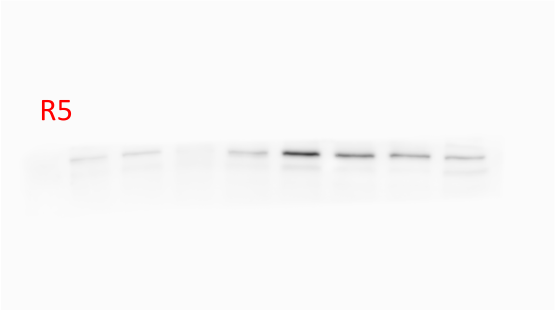

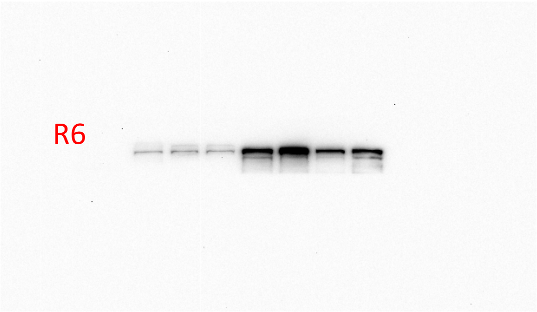

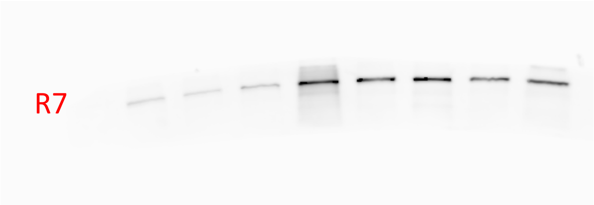

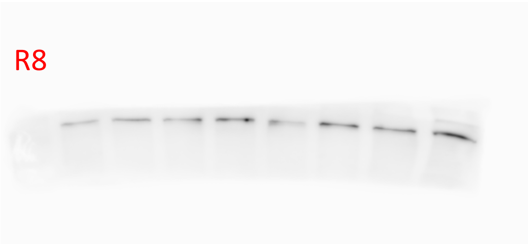

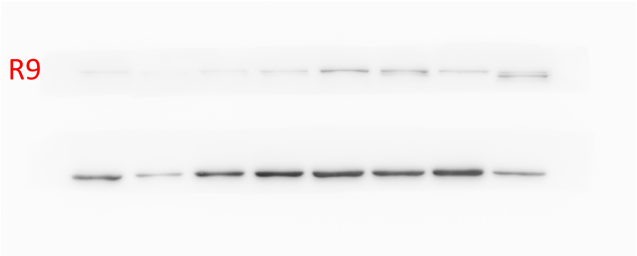

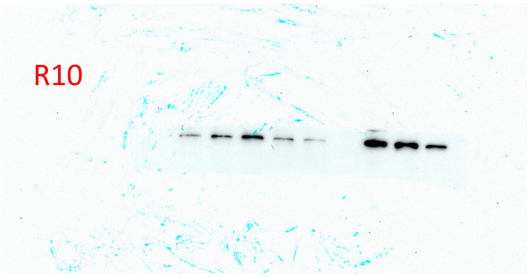

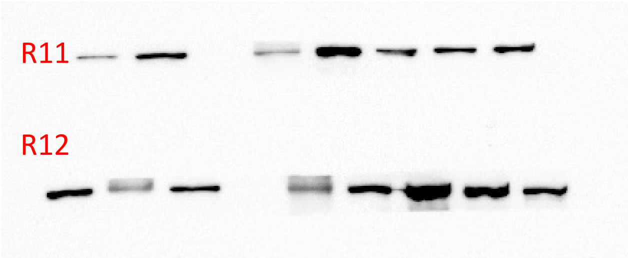

Raw data of Western blots of Rad50

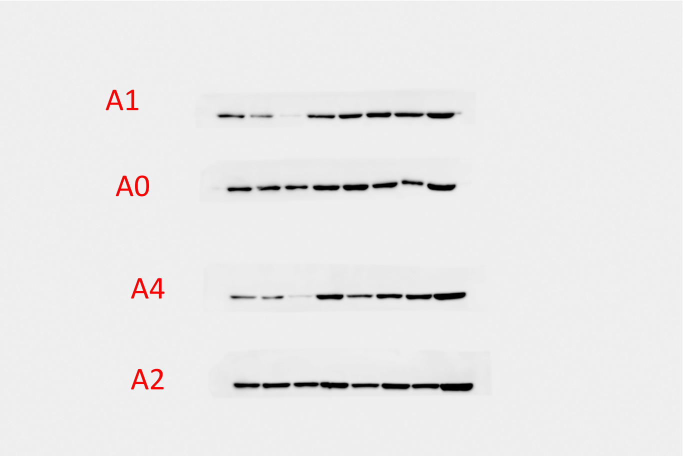

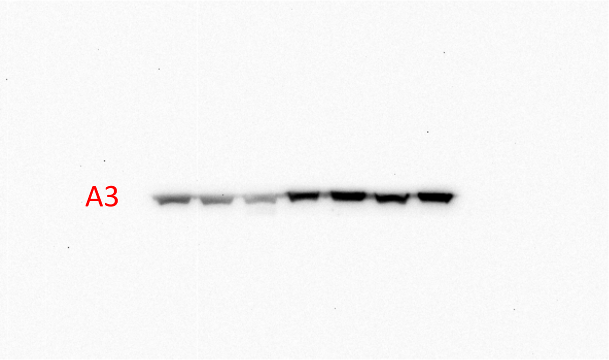

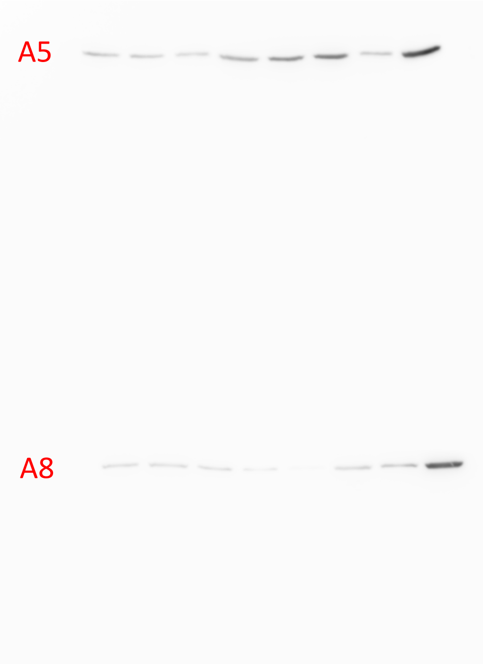

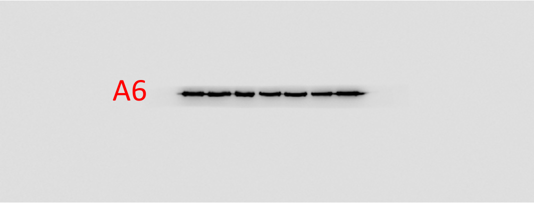

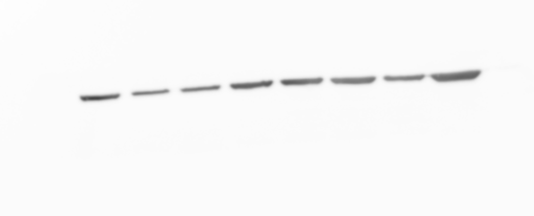

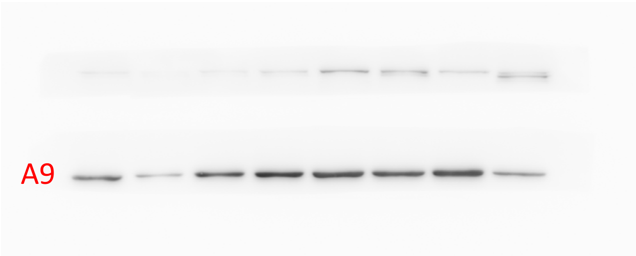

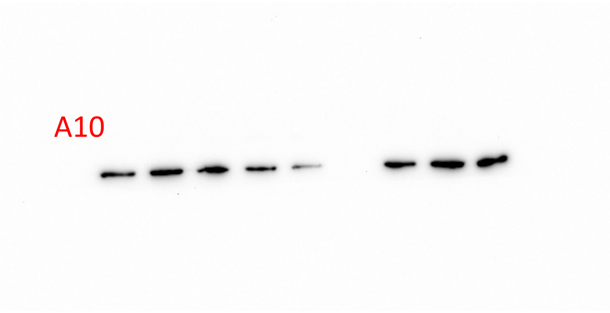

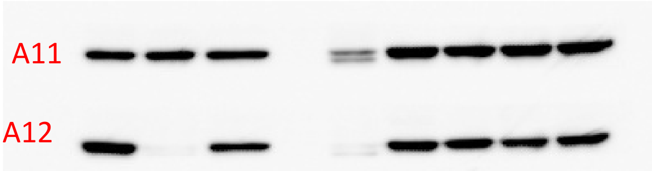

Supplement: Supplementary file 3 — Supplementary Material [file JCMM-25-10961-s001.pdf]
