# Supplementary material for: Rad50 promotes ovarian cancer progression through NF‐κB activation
Source: J Cell Mol Med. 2021 Nov 3;25(23):10961–72. doi: 10.1111/jcmm.17017 (PMC8642684; doi:10.1111/jcmm.17017)
Supplement: Supplementary file 4 — Supplementary Material [file JCMM-25-10961-s003.docx]

**Supplementary Figure Legends**

**Figure S1**. Western blot analysis of Rad50 in the tissues and cell lines. A, Western blot analysis of Rad50 in HGSOC (n=42) compared to normal fimbria (n=31). B, Rad50 expression was analyzed in olaparib resistant ovarian cancer cells in GSE 117765 data. C, Rad50 expression was evaluated using Clinical Proteomic Tumor Analysis Consortium (CPTAC) data. D, Western blot analysis of Rad50 in different cell lines. Expression of Rad50 in established stable cell lines with Rad50 overexpression (Rad50-OVE) or knockdown (shRad50).

Figure S2. Cell cycle analysis of synchronized A2780 cells with Rad50 knockdown. A, Cell cycle analysis showed that Rad50 knockdown led to a decrease in the percentage of cells at G2-M phase compared with control group.

**Figure S3**. Histogram analysis of EMT-related markers by western blot in A2780, SKOV3 and HO8910 cells with Rad50 overexpression or knockdown. A, The histogram reveals ectopic expression of Rad50 in A2780 and HO8910 cells could obviously up-regulate mesenchymal phenotype markers (N-cadherin, Vimentin, Snail, Twist) and down-regulate epithelial phenotype marker (E-cadherin) while Rad50 knockdown upregulated epithelial marker and reduced the levels of mesenchymal markers in A2780 and SKOV3 cells. **P<0.01, ***P<0.001, when compared with the control group.

**Figure S4**. Rad50 promotes tumor metastasis of xenografts. A, Representative images showed much more disseminated peritoneal tumors in Rad50-overexpressed A2780 group compared with the control group.

**Figure S5**. Transwell assays of A2780 and SKOV3 cells with indicated treatments. A, Representative images of transwell assays showed the migration/invasion ability of A2780 cells with or without Rad50 overexpression in the absence or presence of NF-κB inhibitor PDTC. B, Transwell assays of SKOV3 and A2780 cells transfected with indicated combinations of siRNA or vectors.
